# Plasma IgG and IgM autoantibodies to COPT1 as potential biomarkers for detection of non-small cell lung cancer

**DOI:** 10.3389/fimmu.2025.1455095

**Published:** 2025-04-11

**Authors:** Xiaobin Cao, Jing Li, Siyu Liu, Aichen Liu, Lulu Zhang, Fengqi Chen, Yutong Li, Hanke Ma, Wenke Sun, Songyun Ouyang, Liping Dai, Jingjing Liu

**Affiliations:** ^1^ Henan Institute of Medical and Pharmaceutical Sciences & Henan Key Medical Laboratory of Tumor Molecular Biomarkers, Zhengzhou University, Zhengzhou, China; ^2^ Beijing Genomics Institution (BGI) College, Zhengzhou University, Zhengzhou, China; ^3^ Department of Respiratory and Sleep Medicine in the First Affiliated hospital, Zhengzhou University, Zhengzhou, China

**Keywords:** COPT1, autoantibody, non-small cell lung cancer, biomarkers, IgG, IgM

## Abstract

**Background:**

Early diagnosis of lung cancer is crucial for improving patient outcomes. Autoantibodies against tumor-associated antigens (TAAs) found in the plasma can serve as biomarkers for lung cancer detection. Copper transporter 1 (COPT1) is abnormally expressed in several cancers including lung cancer. The purpose of this study is to explore the significance of anti-COPT1 autoantibodies in the clinical diagnosis of non-small cell lung cancer (NSCLC).

**Methods:**

The expression level of COPT1 in NSCLC and normal tissues was analyzed based on TCGA and the Human Protein Atlas (HPA) database. Through enzyme-linked immunosorbent assay (ELISA), the expression levels of anti-COPT1 autoantibodies in plasma samples from normal controls (NC), patients with benign pulmonary nodules (BPN), and patients with NSCLC were detected in the discovery (89 NC and 89 NSCLC) and verification (321 NC, 321 BPN and 321 NSCLC) groups. The ELISA results were verified by western blotting and indirect immunofluorescence experiments.

**Results:**

Based on HPA and TCGA databases, the mRNA and protein levels of COPT1 were higher in NSCLC tissues than in normal tissues. The levels of anti-COPT1-IgG and anti-COPT1-IgM autoantibodies were significantly higher in patients with NSCLC (*P*<0.05). Anti-COPT1-IgG and anti-COPT1-IgM could discriminate NSCLC from NC with area under the curve (AUC) values of 0.733 (95% CI: 0.694-0.771) and 0.679 (95% CI: 0.638-0.720), respectively. Additionally, the combination of anti-COPT1-IgG, anti-COPT1-IgM, and carcinoembryonic antigen (CEA) could enhance the efficacy of NSCLC diagnosis from BPN with increased AUC values.

**Conclusions:**

Our study indicated the potential significance of anti-COPT1-IgG and anti-COPT1-IgM autoantibodies as novel biomarkers for the detection of NSCLC. Furthermore, the combination of anti-COPT1-IgG and anti-COPT1-IgM improved the diagnostic value.

## Introduction

In 2020, lung cancer was projected to account for 2.2 million new cases and 1.8 million deaths, making it the second most common cancer and the leading cause of cancer-related deaths (1). Approximately 85% of lung cancers are non-small cell lung cancer (NSCLC), including lung adenocarcinoma (LUAD) and lung squamous cell carcinoma (LUSC) (2). NSCLC is frequently diagnosed at an advanced stage, with a 5-year survival rate of 14%. Compared to the 5-year survival rate of 83% in patients with stage I NSCLC, early detection plays a crucial role in mitigating the high mortality rate associated with this lethal illness (3).

The early diagnosis of lung cancer is crucial for improving patient outcomes. Bronchoscopy and biopsies are the primary methods used to diagnose lung cancer. Despite being a minimally invasive technique, bronchoscopy may cause discomfort, especially when biopsy samples are obtained from suspected tissues, which can lead to complications. Therefore, monitoring the early development of lung cancer is essential to facilitate timely intervention and enhance the overall prognosis of the disease (4). Prior research has verified that autoantibodies against tumor-associated antigens (TAAs) found in the serum of cancer patients can serve as reliable cancer biomarkers (5, 6).

Autoantibodies are produced early in cancer development as a result of the humoral immune response, which is activated by abnormal expression of TAAs. Tumor-associated autoantibodies are promising candidates for the early detection and treatment of cancer. An increasing number of autoantibodies targeting TAAs have been identified, and their detection has been utilized in research and clinical analysis (7–9). The presence of autoantibodies in cancer patients reflects their increased immune reactivity and enhanced immune surveillance of cancer cells (10). In recent decades, the presence of circulating autoantibodies in the serum of cancer patients has opened up new possibilities for utilizing the immune system as a valuable source of cancer biomarkers (11). Several autoantibodies have been identified in lung cancer and are suggested as potential serum diagnostic markers (12). Among them, p53 autoantibodies in lung cancer have been widely studied, and their prevalence rate accounts for approximately 30% of all lung cancer patients (13). Relevant studies have shown that the production of autoantibodies precedes the appearance of symptoms and the diagnosis of NSCLC (14). Therefore, the detection and characterization of autoantibodies are likely to provide new insights into the complex biology of the humoral response in lung cancer and will have an important impact on the clinical management of lung cancer patients in the future.

Copper (Cu) transporter 1 (COPT1), a member of solute carrier family 31 (SLC31A1), is considered the key component of Cu absorption by mammalian cells and tissues (15). *COPT1* has recently been identified as a regulatory gene for copper, and high expression levels of COPT1 can lead to a types of cell death known as cuproptosis (16). The function of COPT1 in tumor progression has also been investigated. Blocking the COPT1‐copper axis diminishes AKT signaling and reduces tumorigenesis (17). The COPT1-MEK-DNMT1-miR-124 feedback loop promotes pancreatic cancer progression (18). Moreover, COPT1 enhances chemoresistance by constraining CPT1A-mediated fatty acid oxidation process in ER-positive breast cancer (19). Expression levels of COPT1 have been shown to be elevated in breast invasive carcinoma, esophageal carcinoma, glioblastoma multiforme, and gastric adenocarcinoma compared with those in normal tissues (20). Li et al. found that COPT1 may be a promising biomarker for diagnosis and prognosis and a predictor of drug response in breast cancer (21). COPT1 has been identified as a TAA overexpressed in NSCLC patients, suggesting its potential as a biomarker for clinical diagnosis (20). TAAs exhibit significant limitations as biomarkers, including low expression in plasma and short circulation time. In contrast, autoantibodies against TAAs have the advantages of high stability, specificity and sensitivity. However, no relevant studies have investigated whether there is an autoantibody response to COPT1 in patients with NSCLC. The value of anti-COPT1 autoantibodies for in the detection of NSCLC has not yet been investigated.

In this study, we aimed to explore the expression levels of anti-COPT1 autoantibodies in the plasma of NSCLC patients, and their potential as biomarkers for the clinical diagnosis of NSCLC.

## Materials and methods

### Study design

**Figure 1:**
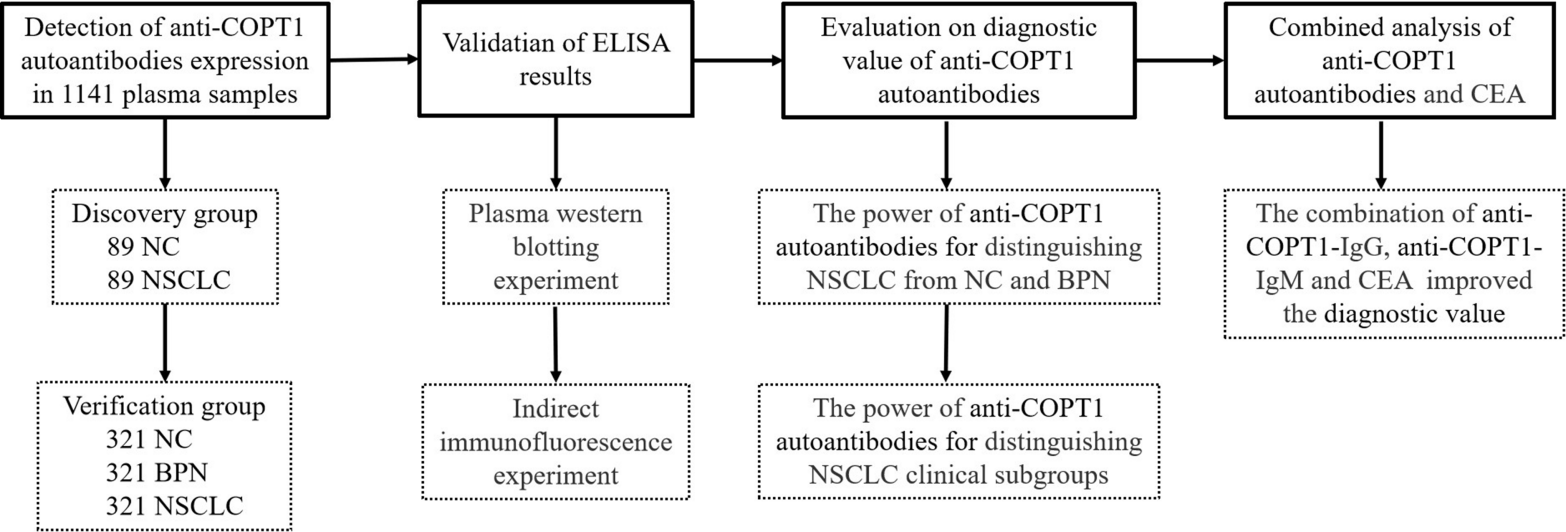
The overall study design. NSCLC, Non-small Cell Lung Cancer; NC, Normal Control; BPN, Benign Pulmonary Nodule; ELISA, Enzyme-linked Immunosorbent assay.

The study design is illustrated in **Figure 1**. The plasma samples were divided into discovery and verification groups. First, the expression levels of anti-COPT1 autoantibodies were detected in both the discovery and verification groups using enzyme-linked immunosorbent assay (ELISA). Subsequently, western blotting and indirect immunofluorescence were performed to verify the experimental results of ELISA. Then, the diagnostic efficacy of anti-COPT1 autoantibodies in distinguishing NSCLC was analyzed. Finally, we evaluated the potential of anti-COPT1 autoantibodies in combination with carcinoembryonic antigen (CEA) as biomarkers for NSCLC detection.

### Eligibility criteria

The samples were collected at the First Affiliated Hospital of Zhengzhou University (Zhengzhou, China). The sample cohort consisted of patients with NSCLC, patients with benign pulmonary nodules (BPN) and normal control controls (NC). There were 89 NSCLC and 89 NC in the discovery group, and 321 NSCLC, 321 BPN and 321 NC in the verification group. Among 321 NSCLC in the verification group, there were 174 early NSCLC and 89 advanced NSCLC. All patients involved in this study strictly complied with the diagnostic standards. NC individuals without any history of disease or tumors underwent physical examinations in the same hospital. The NSCLC, BPN and NC individuals were matched in a 1:1:1 ratio according to sex and age (± 5 years). This study was approved by the Ethics Committee of the First Affiliated Hospital of Zhengzhou University, and informed consent was obtained from all participants.

### Sample collection

Blood samples were obtained using 5 ml vacuum blood collection tubes containing EDTA-K2 anticoagulant. Venous blood obtained from the elbow on an empty stomach was mixed with an anticoagulant by inverting the blood collection tube 5-8 times. Subsequently, the plasma was separated by centrifugation at 3000 r/min at 37°C for 5 min. The upper plasma was carefully extracted using a disposable straw and transferred to a 1.5 ml tube. Detailed information regarding sample numbers, clinical characteristics, and CEA expression level was noted before promptly storing the plasma samples in an ultra-low temperature freezer at -80°C. To prevent damage, the required samples were thawed in the refrigerator at 4°C prior to use, mitigating the need for repetitive freezing and thawing of the original tube.

### Enzyme-linked immunosorbent assay

In this study, the expression of anti-COPT1 autoantibodies in the plasma with ELISA was detected using the recombinant COPT1 protein purchased from Cloud-Clone Corp (No.3AA85A2E08, Wuhan, China). The specific experimental procedure involved the coating of the recombinant COPT1 at a concentration of 0.125 μg/ml on a 96-well ELISA plate, followed by 1% BSA coating. After coating overnight, the plates were sealed at 37°C for 2 hours with 2% BSA solution. The blocking solution was discarded and the primary antibody was incubated at 37°C for 1 h. Finally, the secondary antibody was incubated at 37°C for 1 h. Plasma samples were diluted at a ratio of 1:100 to serve as the primary antibody. Goat anti-human IgG or IgM antibody labeled with horseradish peroxidase (HRP) was used as the secondary antibody. Anti-human IgG antibody was diluted at a ratio of 1:10000 and anti-human IgM antibody at a ratio of 1:5000. Each plate was equipped with two duplicate wells for quality control purposes and two blank controls to ensure consistency and precision of the optical density (OD) values obtained from all wells. Specific Binding Index (SBI) = (OD value of sample to be tested-OD value of blank hole)/(OD value of quality control sample-OD value of blank hole). The blood samples of 100 normal controls were fully and equally mixed for quality control which is used to eliminate the errors between enzyme-labeled plates and represent the general level of the population.

### Western blotting

Western blotting was performed to confirm the expression of anti-COPT1 autoantibodies in plasma. The recombinant COPT1 protein was loaded onto a 10% SDS-PAGE gel at a total loading amount of 3 μg. Subsequently, the proteins were subjected to electrophoresis at 150 V for 60 min and transferred onto a PVDF membrane at 100 V for 90 min. After sealing for 2 h, the membrane was then cut into 10 strips of uniform size. Each strip was incubated with different primary antibodies. The mouse anti-COPT1 monoclonal antibody (67221-1-Ig, Proteintech, Wuhan, China) was diluted 1:1000 to serve as a positive control primary antibody for one strip. Nine different plasma samples (three LC, three BPN and three NSCLC) were diluted of 1:100 as the primary antibodies for the other nine strips, respectively. Finally, the secondary antibodies, goat anti-mouse and sheep anti-human IgG antibodies labeled with HRP, were utilized to detect specific protein bands indicative of presence of the anti-COPT1 autoantibodies.

### Indirect immunofluorescence

To begin the experiment, A549 cells in logarithmic growth period were digested, centrifuged, and counted. The cell density was adjusted to 1×10^5^ cells/ml. Subsequently, 20 μl of the diluted cell suspension was added to a cell slide, which was then placed into a 24-well plate. Once the cells adhered to the wall, 380 μl of complete culture medium was added to each well. Fixation was then performed using 4% paraformaldehyde for 20 min, followed by blocking with 0.5% BSA solution for 1 h. Next, the anti-COPT1 monoclonal antibody, one NSCLC and one NC plasma were served as primary antibodies, while goat anti-mouse IgG and sheep anti-human antibodies labeled with FITC were used as secondary antibodies. Finally, the fluorescence intensity of the cell slides incubated with different antibodies was observed using a fluorescence microscope.

### Statistical analysis

Statistical analysis and visualization of experimental results were conducted using SPSS 21.0 and GraphPad 8.0. PASS 15.0 software was used to calculate the required sample size. Previous studies have showed that the diagnostic sensitivity of autoantibodies is approximately 10%-30%, and the specificity is 70%-80% (22). The allowable error δ is set to 0.05-0.1, and the confidence level is 1-α=0.95. The calculation formula is n=(Z_α_/δ)^2^ (1-*P*) *P*. According to PASS 15.0, the sample number for NSCLC was 86-333 and the sample number for BPN and NC was 66-333. The expression levels of anti-COPT1 autoantibodies in NSCLC, BPN and NC were compared using the Mann-Whitney U test.

Receiver operating characteristic (ROC) curve analysis was performed to evaluate the differential diagnostic value AUC, 95% confidence interval (95% CI), sensitivity, specificity, and Youden’s index (YI) of anti-COPT1 autoantibodies for NSCLC and its various clinical features. AUC value was used to measure the diagnostic ability. AUC<0.5 shows no diagnostic ability. AUC>0.7 indicates certain diagnostic ability and AUC>0.8 indicates good diagnostic ability (23). The maximum YI is a comprehensive index of sensitivity and specificity that can help determine the best diagnostic threshold and compare the accuracy of different tests. Here, the maximum YI was used as the standard for the best threshold to identify true positives and avoid false positives.

Additionally, the positive rates of COPT1 autoantibodies in different clinical features of NSCLC patients were compared using the chi-square test. Pairwise comparisons between the two groups were further analyzed using the Wilcoxon test with Bonferroni adjustment. Logistic regression analysis was used to construct the combined models. The optimal cutoff value, which was utilized to determine the positive rate, was set at the SBI value with the largest YI. Statistical significance was determined when the AUC was greater than 0.5 and *P* was less than 0.05.

## Results

### COPT1 is highly expressed in patients with NSCLC

**Figure 2:**
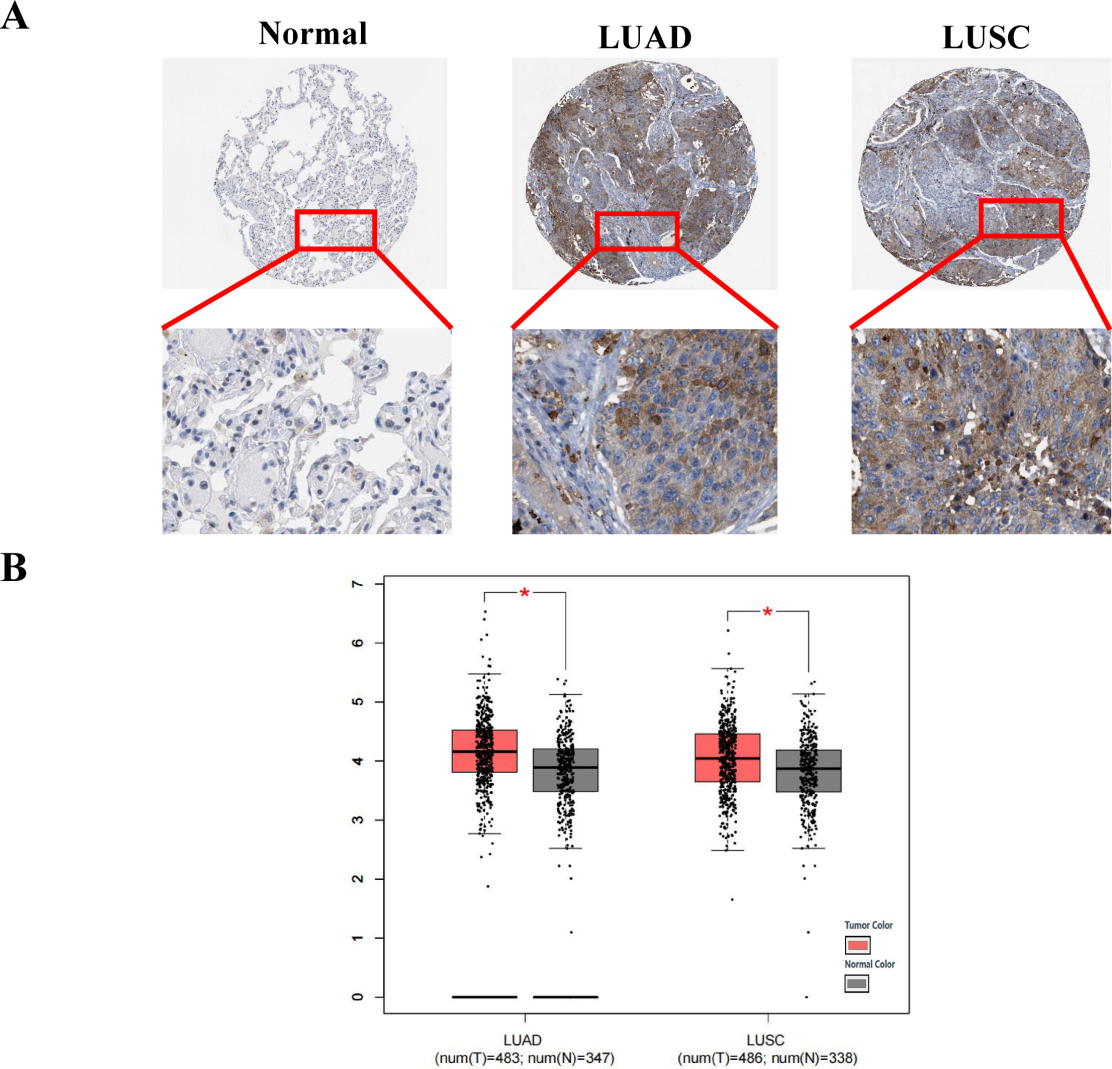
COPT1 was highly expressed in patients with non-small cell lung cancer. **(A)** IHC staining images of COPT1 from HPA database in normal tissues and NSCLC tissues. **(B)** The expression of COPT1 in normal tissues, LUAD and LUSC from TCGA and GTEx databases. HPA, The Human Protein Atlas; LUAD, Lung adenocarcinoma; LUSC, Lung squamous cell carcinoma; T, Tumor; N, Normal; *P<0.05.

According to data from the Human Protein Atlas (HPA) online database, the protein level of COPT1 was higher in patients with NSCLC than in healthy individuals (**Figure 2A**). COPT1 was not expressed in normal lung vacuole cells but was moderately (2/6) or weakly (1/6) in LUAD, and moderately expressed (2/5) or weakly (1/5) expressed in LUSC by HPA database (**Figure 2A**). Furthermore, analyses of the TCGA and GTEx data revealed that the mRNA expression levels of COPT1 were elevated in patients with LUSC and LUAD (*P*< 0.05, **Figure 2B**). These findings collectively emphasized that the expression of COPT1 was significantly elevated in NSCLC patients compared with that in NC.

### The expression level of anti-COPT1 autoantibodies was preliminarily detected in the discovery group

**Figure 3:**
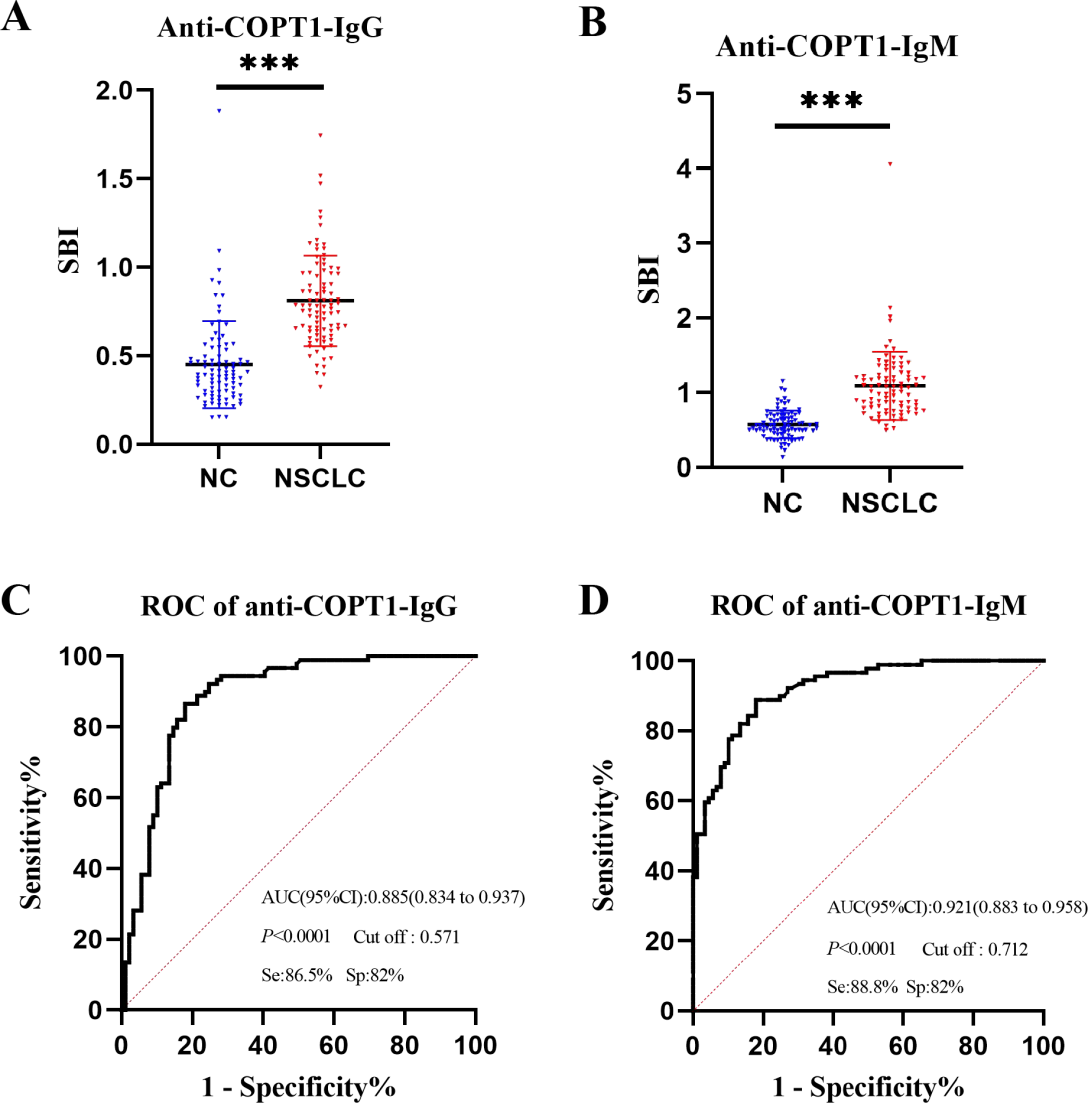
Expression level of anti-COPT1 autoantibodies in the discovery group. **(A, B)** Expression levels of anti-COPT1-IgG and anti-COPT1-IgM autoantibodies in 89 NSCLC patients and 89 normal controls, respectively. **(C, D)** ROC curve, AUC, specificity and sensitivity of anti-COPT1-IgG and anti-COPT1-IgM autoantibodies in 89 NSCLC patients and 89 NC, respectively. Se, Sensibility; Sp, Specificity. ***P<0.001.

The expression levels of anti-COPT1-IgG and anti-COPT1-IgM in the plasma of the 89 NSCLC and 89 NC in the discovery group were detected by ELISA. The demographic information of the participants is presented in **Table 1**. ELISA results revealed that the patients with NSCLC exhibited significantly elevated levels of anti-COPT1-IgG and anti-COPT1-IgM compared with NC (**Figures 3A, B**). The AUCs of anti-COPT1-IgG and anti-COPT1-IgM autoantibodies for distinguishing NSCLC from NC were 0.885 (95% CI: 0.834-0.937, sensitivity=86.5%, specificity=82.0%) and 0.921 (95% CI: 0.883-0.958, sensitivity=88.8%, specificity=82.0%), respectively (**Figures 3C, D**). These results suggested that NSCLC patients have higher levels of anti-COPT1 autoantibodies expression.

**Table 1:** Clinical characteristics of NSCLC and NC in the discovery group.

Variables	NSCLC	NC	P
	(n=89)	(n=89)	
Age (year, %)			
≤55y	32(35.9)	36 (40.4)	>0.05
>55y	57(64.1)	53 (59.6)	
Mean ± SD	60 ± 10	58 ± 9	
Gender, n (%)			
Male	51(57.3)	49(55.0)	>0.05
Female	38(42.7)	40(45.0)	
Histology, n (%)			
LUAD	61(68.5)		
LUSC	16(17.9)		
Other NSCLC	12(13.6)		
Smoking, n (%)			
Yes	29(32.5)		
No Unknown	57(64.0) 3 (3.5)		
Drinking, n (%)			
Yes	13(14.5)		
No Unknown	73(82.0) 3 (3.5)		
Lymph node Metastasis, n (%)			
Yes	46(51.6)		
No	42(47.1)		
Unknown	1(1.3)		
Distant Metastasis, n (%)			
Yes	45(50.5)		
No	37(41.6)		
Unknown	7(7.9)		
Clinical stage, n (%)			
I	31(34.8)		
II	11(12.4)		
III	0(0)		
IV	47(52.8)		
Unknown	0(0)		

*NSCLC*, Non-small cell lung cancer; *NC*, Normal controls; *SD*, standard deviation.

### The expression level of anti-COPT1 autoantibodies was verified in the verification group

**Figure 4:**
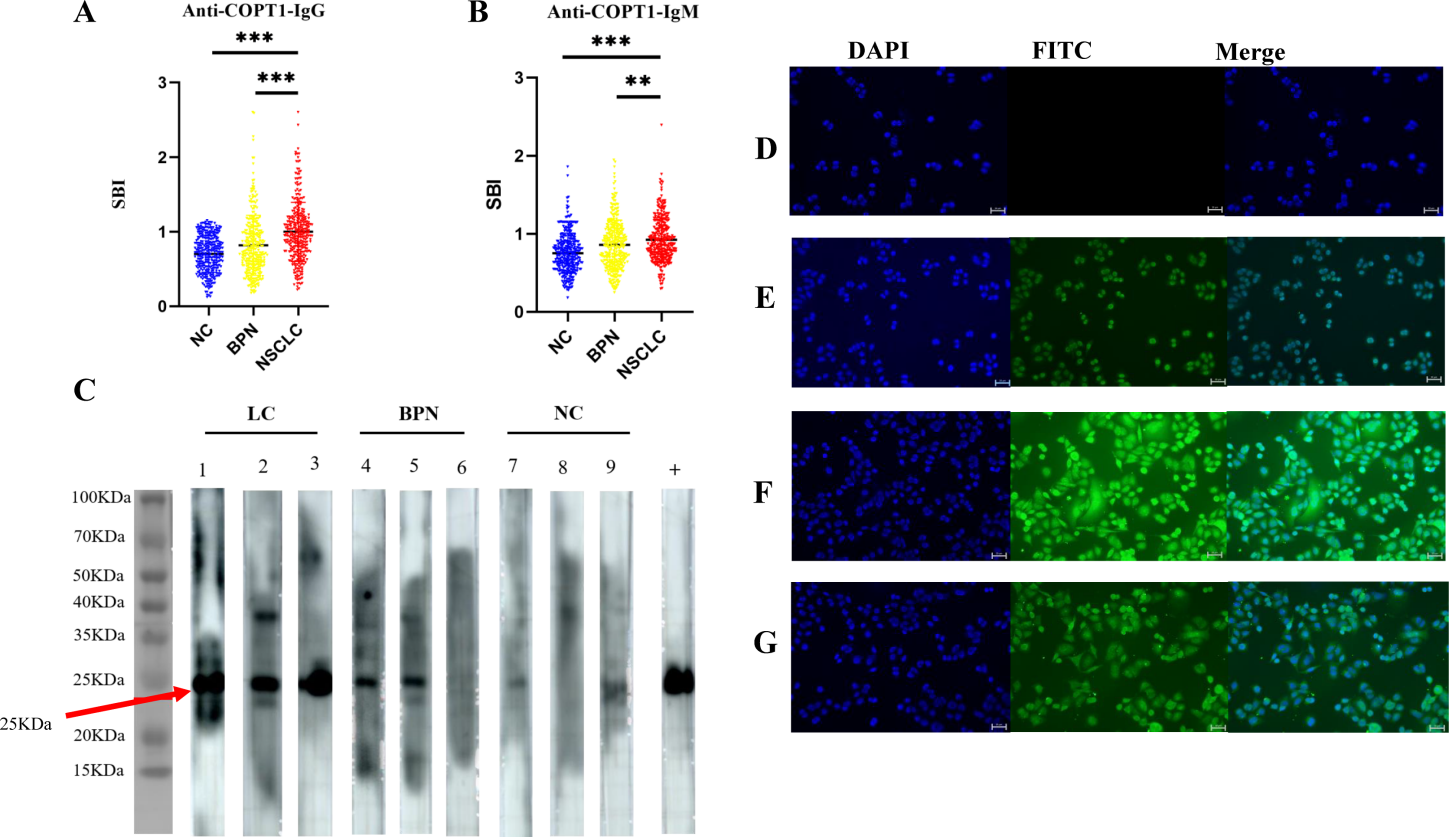
Validation of anti-COPT1 autoantibodies expression in the verification group. **(A, B)** The expression of anti-COPT1-IgG and anti-COPT1-IgM autoantibodies in 321 NSCLC, 321 BPN and 321 NC were detected by ELISA. **(C)** The expression of anti-COPT1 autoantibodies in the plasma samples of 3 NSCLC, 3 BPN and 3 NC was detected by western blotting. Lane +, The anti-COPT1 monoclonal antibody used as the positive control. **(D–G)**, Indirect immunofluorescence detection of anti-COPT1 autoantibodies in the plasma samples of NSCLC and NC. **(D)** The blank control, PBS a s primary antibody incubation; **(E)** The positive control, an anti-COPT1 monoclonal antibody as primary antibody; **(F)** The plasma of NSCLC as primary antibody; **(G)** NC plasma as primary antibody. **P<0.01; ***P<0.001.

The expression levels of anti-COPT1 autoantibodies in plasma were further detected in the verification group (321 NSCLC, 321 BPN and 321 NC) to validate previous findings and enhanced the robustness of the results (**Table 2**). The results showed that anti-COPT1-IgG and anti-COPT1-IgM levels were elevated in NSCLC patients compared with those in BPN and NC (**Figures 4A, B**).

**Table 2:** Clinical characteristics of NSCLC, BPN and NC in the verification group.

Variables	NSCLC	BPN	NC	P
	(n=321)	(n=321)	(n=321)	
Age (year, %)				
≤55y	164(51.1)	165(51.4)	170(53.0)	>0.05
>55y	157(48.9)	156(48.6)	151(47.0)	
Mean ± SD	56 ± 11	55 ± 12	56 ± 11	
Gender, n (%)				
Male	199(62.0)	200(62.3)	204(63.5)	>0.05
Female	122(38.0)	121(37.7)	117(36.5)	
Histology, n (%)				
LUAD	227(70.7)			
LUSC	46(14.3)			
Other NSCLC	48(15.0)			
Smoking, n (%)				
Yes	114(35.5)	42(13.1)		
No Unknown	207(64.5) 0(0)	267(83.1) 12(3.8)		
Drinking, n (%)				
Yes	85(26.5)	86(26.8)		
No Unknown	236(73.5) 0(0)	233(72.6) 2(0.6)		
Lymph node Metastasis, n (%)				
Yes	97(30.2)			
No	185(57.6)			
Unknown	39(12.2)			
Distant Metastasis, n (%)				
Yes	40(12.5)			
No	228(71.0)			
Unknown	53(16.5)			
Clinical stage, n (%)				
I	158(49.2)			
II	16(5.0)			
III	53(16.5)			
IV	36(11.2)			
Unknown	58(18.1)			

*NSCLC*, Non-small cell lung cancer; *BPN*, Benign pulmonary nodules; *NC*, Normal controls; *SD*, standard deviation.

The expression levels of anti-COPT1 autoantibodies in the plasma were confirmed by western blotting. Three plasma samples from NSCLC patients with high anti-COPT1 autoantibody expression levels, three plasma samples from BPN patients, and three plasma samples from NC were selected. To verify the ELISA results, a purchased monoclonal antibody of COPT1 was utilized as a positive control. The findings revealed that the NSCLC plasma with a high level of anti-COPT1 autoantibodies in ELISA also showed strong reactivity in western blotting analysis (**Figure 4C**). Western blotting results confirmed the occurrence of immunoreactivity to COPT1 in the plasma.

Using IIF in A549 cells with high COPT1 expression (**Supplementary Figure 1A**), we detected the expression of anti-COPT1 autoantibodies in both NSCLC and NC to confirm the anti-COPT1 autoantibody response (**Figures 4D–G**). Our results confirmed the presence expression of anti-COPT1 autoantibodies in the plasma.

### The diagnostic value of anti-COPT1 autoantibodies was evaluated as biomarkers to distinguish NSCLC from NC

**Figure 5:**
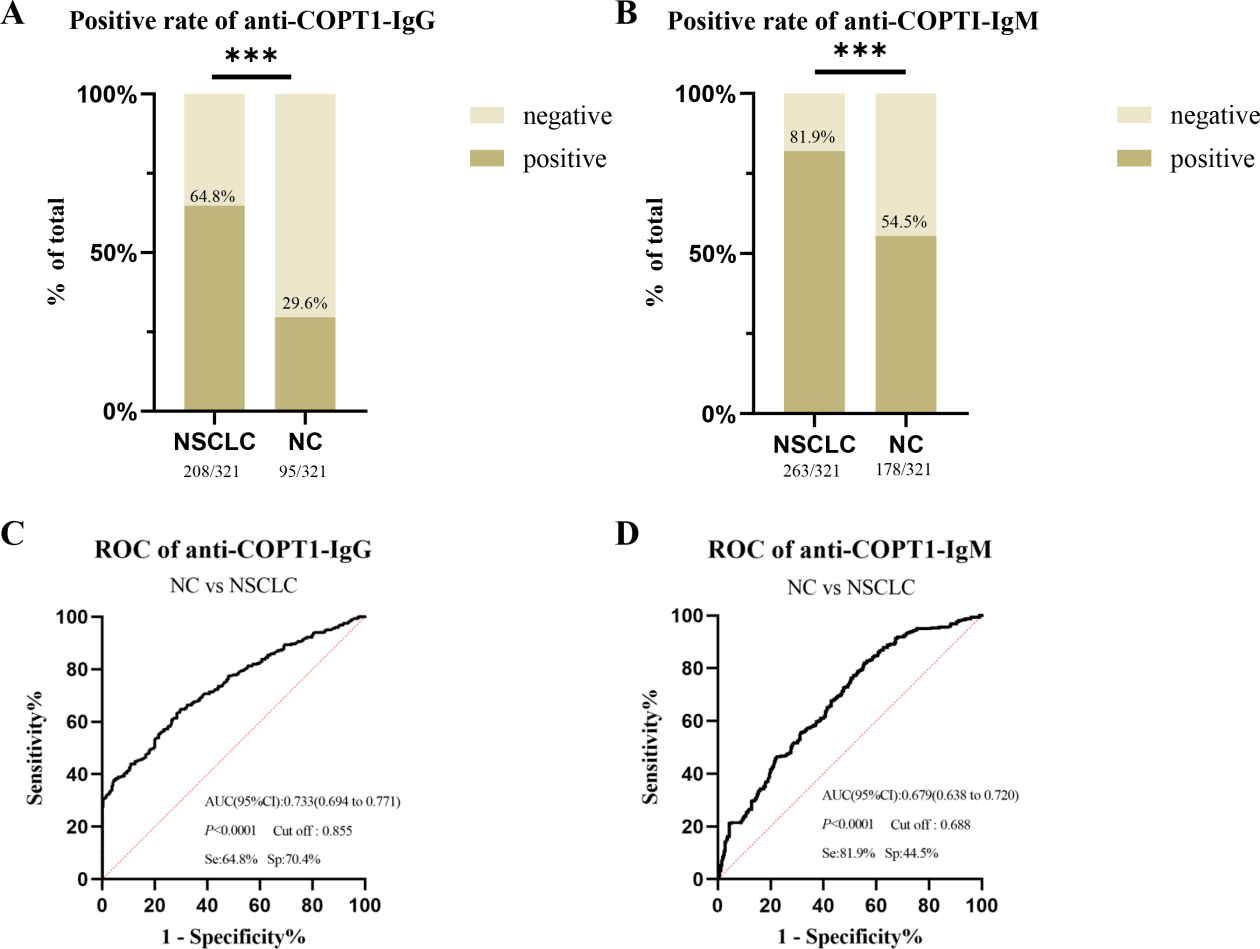
The diagnostic value of anti-COPT1 autoantibodies. **(A, B)** the positive rates of anti-COPT1-IgG and anti-COPT1-IgM autoantibodies in NSCLC and NC, respectively. **(C, D)** ROC curve, AUC, sensitivity and specificity of anti-COPT1-IgG and anti-COPT1-IgM autoantibodies in 321 NSCLC and 321 NC, respectively. Se, Sensibility; Sp, Specificity. ***P<0.001.

The positive rates of anti-COPT1-IgG and anti-COPT1-IgM in NSCLC were higher than those in NC in the verification group (**Figures 5A, B**). When distinguishing NSCLC from NC, the AUC values of anti-COPT1-IgG and anti-COPT1-IgM were 0.733 (95% CI: 0.694-0.771, sensitivity=64.8%, specificity=70.4%) and 0.679 (95% CI: 0.638-0.720, sensitivity=81.9%, specificity=44.5%), respectively (**Figures 5C, D**). These results showed that anti-COPT1 autoantibodies can effectively distinguish NSCLC from NC.

### Anti-COPT1 autoantibodies could distinguish LUSC and LUAD from NC

**Figure 6:**
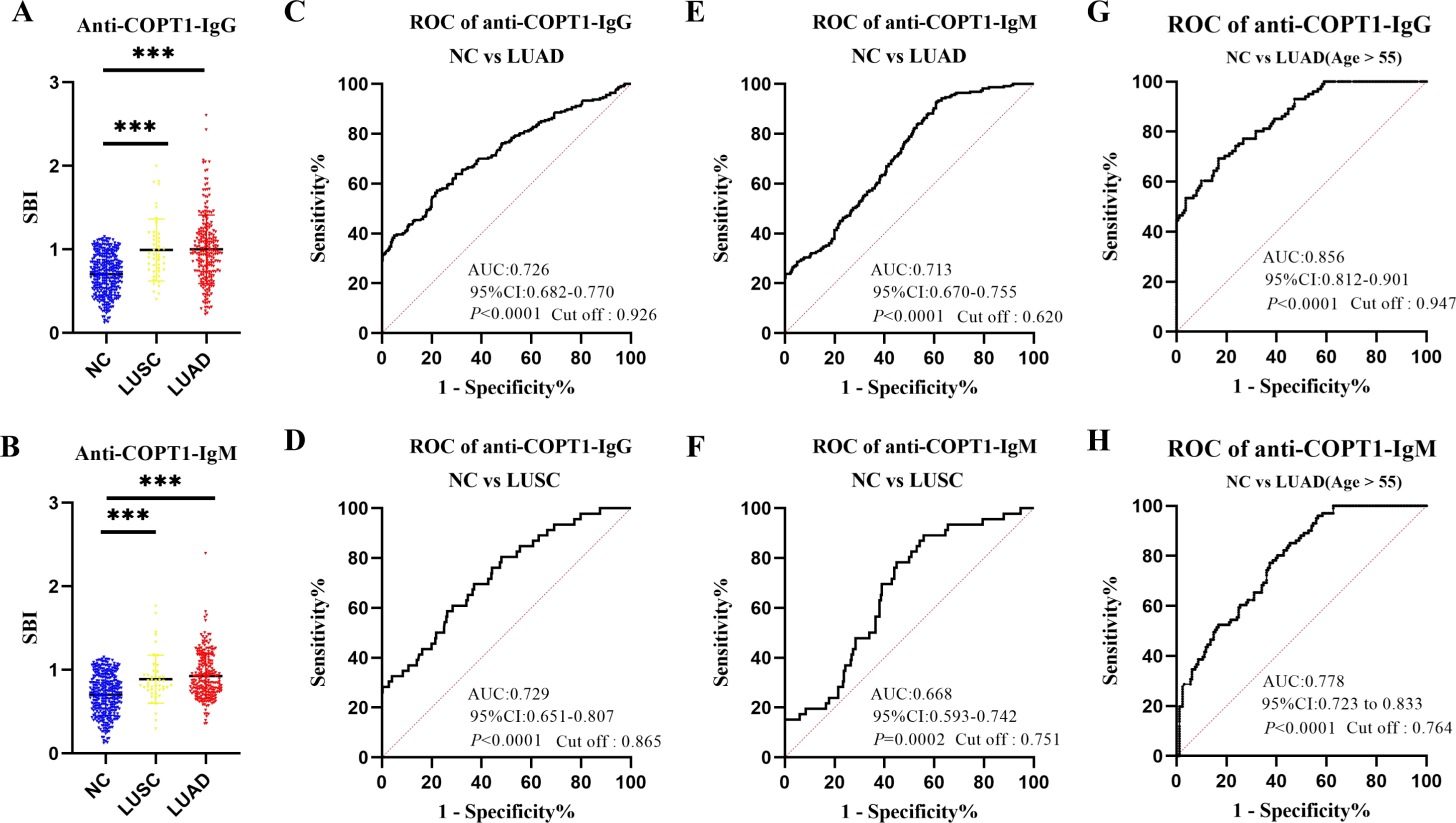
Anti-COPT1 autoantibodies can distinguish LUAD and LUSC from NC. **(A, B)** The expression of anti-COPT1-IgG and anti-COPT1-IgM in LUSC, LUAD and NC, respectively. **(C, D)** ROC curve analysis of anti-COPT1-IgG autoantibody to distinguish LUAD and LUSC patients from NC, respectively. **(E, F)** ROC curve analysis of anti-COPT1-IgM autoantibody to distinguish LUAD and LUSC patients from NC, respectively. **(G, H)** ROC curve analysis of anti-COPT1-IgG and anti-COPT1-IgM autoantibody to distinguish LUAD with age>55 from NC, respectively. ***P<0.001.

As LUSC and LUAD are the most common subtypes of NSCLC, we evaluated the diagnostic efficacy of anti-COPT1 autoantibodies in these two subtypes of NSCLC. The verification group comprised 227 patients with LUAD and 46 patients with LUSC. Both anti-COPT1-IgG and anti-COPT1-IgM autoantibodies exhibited higher expression levels in LUAD and LUSC than in NC. Interestingly, no statistically significant differences were observed in the expression levels of anti-COPT1-IgG and anti-COPT1-IgM autoantibodies between LUAD and LUSC (*P*>0.05) (**Figures 6A, B**). The AUC values for anti-COPT1-IgG autoantibodies in LUAD and LUSC were 0.726 (95% CI: 0.682-0.770) and 0.729 (95% CI: 0.651-0.807), respectively (**Figures 6C, D**). The AUC values of the anti-COPT1-IgM autoantibodies for distinguishing LUAD and LUSC from NC were 0.713 (95% CI: 0.670-0.755) and 0.668 (95% CI: 0.593-0.742), respectively (**Figures 6E, F**). Pairwise comparisons of different subgroups of LUAD stratified by age, gender, smoking and drinking status were performed (**Supplementary Figure 2**, **Figures 6G, H**). For individuals aged >55 years, the increased AUC values for anti-COPT1-IgG and anti-COPT1-IgM were 0.856 (95% CI: 0.812-0.901) and 0.778 (95% CI: 0.723-0.833), respectively. Therefore, anti-COPT1 autoantibodies can be used to distinguish LUAD and LUSC from NC.

### Anti-COPT1 autoantibodies can distinguish NSCLC clinical subgroups from NC

**Figure 7:**
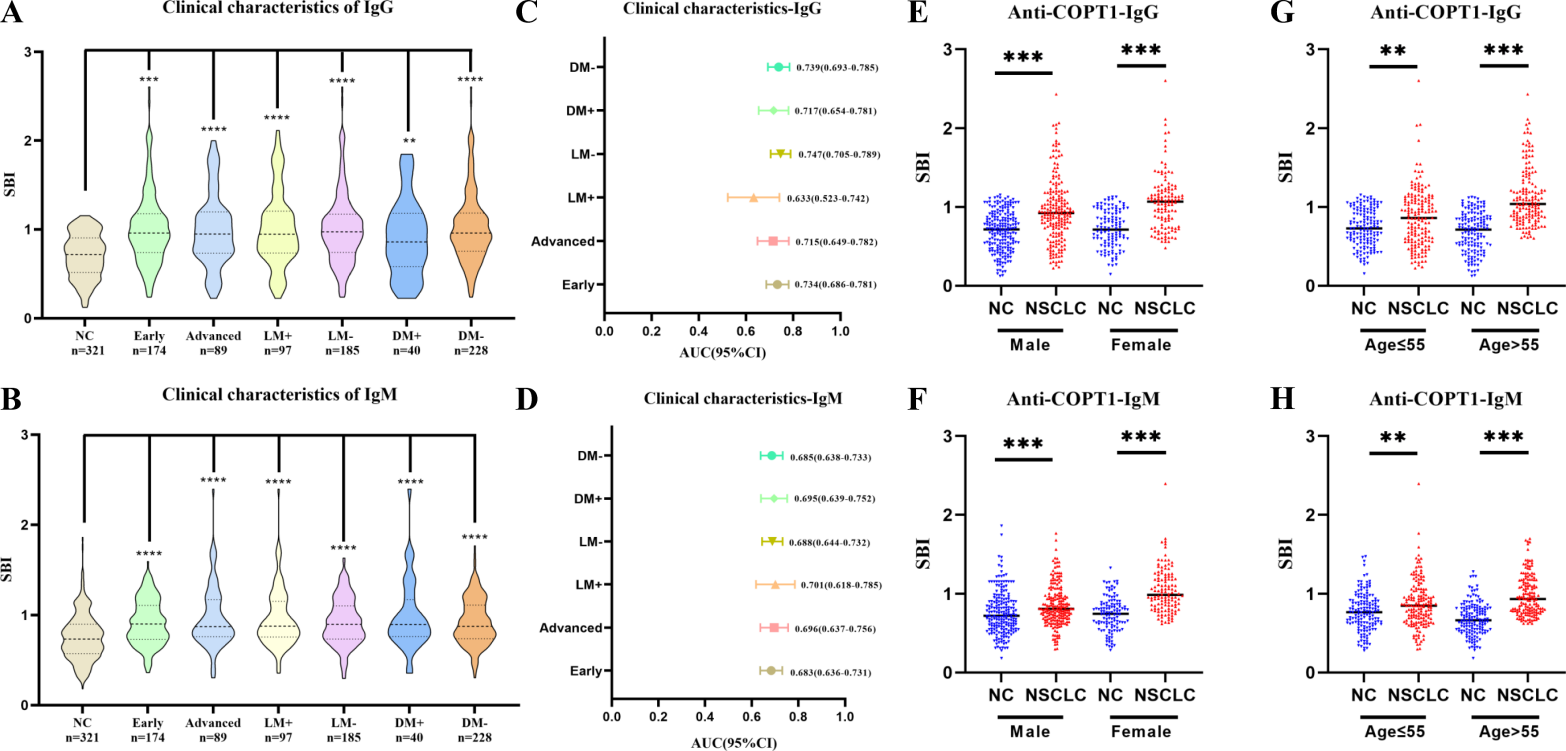
Expression of anti-COPT1 in different clinical subgroups and NC. **(A, B)** Expression of anti-COPT1-IgG and anti-COPT1-IgM autoantibodies in different clinical subgroups, respectively. **(C, D)** AUC and 95% CI of anti-COPT1 autoantibodies in different clinical subgroups to distinguish NSCLC with different clinical features from NC. **(E–H)** The expression of anti-COPT1-IgG and anti-COPT1-IgM autoantibodies stratified by age and gender. Early, Patients with early NSCLC (clinical stage I&II); Advanced, Patients with advanced NSCLC (clinical stage III&IV); LM+, Lymph node metastasis positive; LM-, Lymph node metastasis negative; DM+, Distant metastasis positive; DM-, Distant metastasis negative; **P<0.01, ***P<0.001, ****P<0.0001.

To further study the expression level of anti-COPT1 autoantibodies in different clinical subgroups, NSCLC was divided according to clinical stage, lymph node metastasis, distant metastasis, smoking and drinking. There were no significant differences in the expression levels of anti-COPT1-IgG and anti-COPT1-IgM between the different subgroups (*P*>0.05) (**Supplementary Figures 1B, C**). In every subgroup, both anti-COPT1-IgG and anti-COPT1-IgM expression levels were higher in NSCLC (*P*<0.05) (**Figures 7A, B**). The AUC range for anti-COPT1-IgG in various clinical subgroups was 0.633-0.747 (**Figure 7C**, **Supplementary Figure 3**). There was no significant difference in the expression levels of anti-COPT1 autoantibodies between early and advanced NSCLC (**Supplementary Figures 1B, C**), indicating that the anti-COPT1 autoantibodies did not increase with tumor stage progression. The AUC value of anti-COPT1-IgG for the identification of early stage NSCLC from NC was 0.734 (95% CI: 0.686-0.781, sensitivity=63.8%, specificity=71.7%), which was higher than that for NSCLC with late stage (AUC=0.715, 95% CI: 0.649-0.782, sensitivity=38.2%, specificity=90.6%, **Figure 7C**, **Supplementary Figures 3A, B**). For the anti-COPT1-IgM autoantibody, the AUC range in different clinical subgroups was 0.683-0.701 (**Figure 7D**, **Supplementary Figure 4**). The anti-COPT1-IgM autoantibody could discriminate early NSCLC from NC, with an AUC of 0.683 (95% CI: 0.636-0.731, sensitivity=49.4%, specificity=77.9%) (**Figure 7D**, **Supplementary Figure 4A** **).** These results indicated the potential of anti-COPT1 autoantibodies as biomarkers for the detection of early NSCLC.

Pairwise comparisons of the different subgroups according to age and gender were performed. In the different subgroups, anti-COPT1 autoantibody levels were higher in NSCLC than in NC (**Figures 7E–H**). For female individuals, the AUC values of anti-COPT1-IgG and anti-COPT1-IgM autoantibodis for distinguishing NSCLC from NC were 0.801 (95% CI: 0.747-0.855, sensitivity=53.3%, specificity=94.1%) and 0.808 (95% CI: 0.755-0.861, sensitivity=64.8%, specificity=82.2%), respectively (**Supplementary Figure 4**). For individuals aged >55 years, the AUCs of anti-COPT1-IgG and anti-COPT1-IgM were 0.851 (95% CI: 0.810-0.891, sensitivity= 77.1%, specificity=74.2%) and 0.818 (95% CI: 0.773-0.864, sensitivity=95.5%, specificity=51.0%), respectively, which were higher than those for individuals aged ≤55 years (**Supplementary Figures 3**, **4**). These expression of anti-COPT1 autoantibodies and the diagnostic accuracy were influenced by age and gender.

### Anti-COPT1 autoantibodies can effectively distinguish NSCLC from BPN

**Figure 8:**
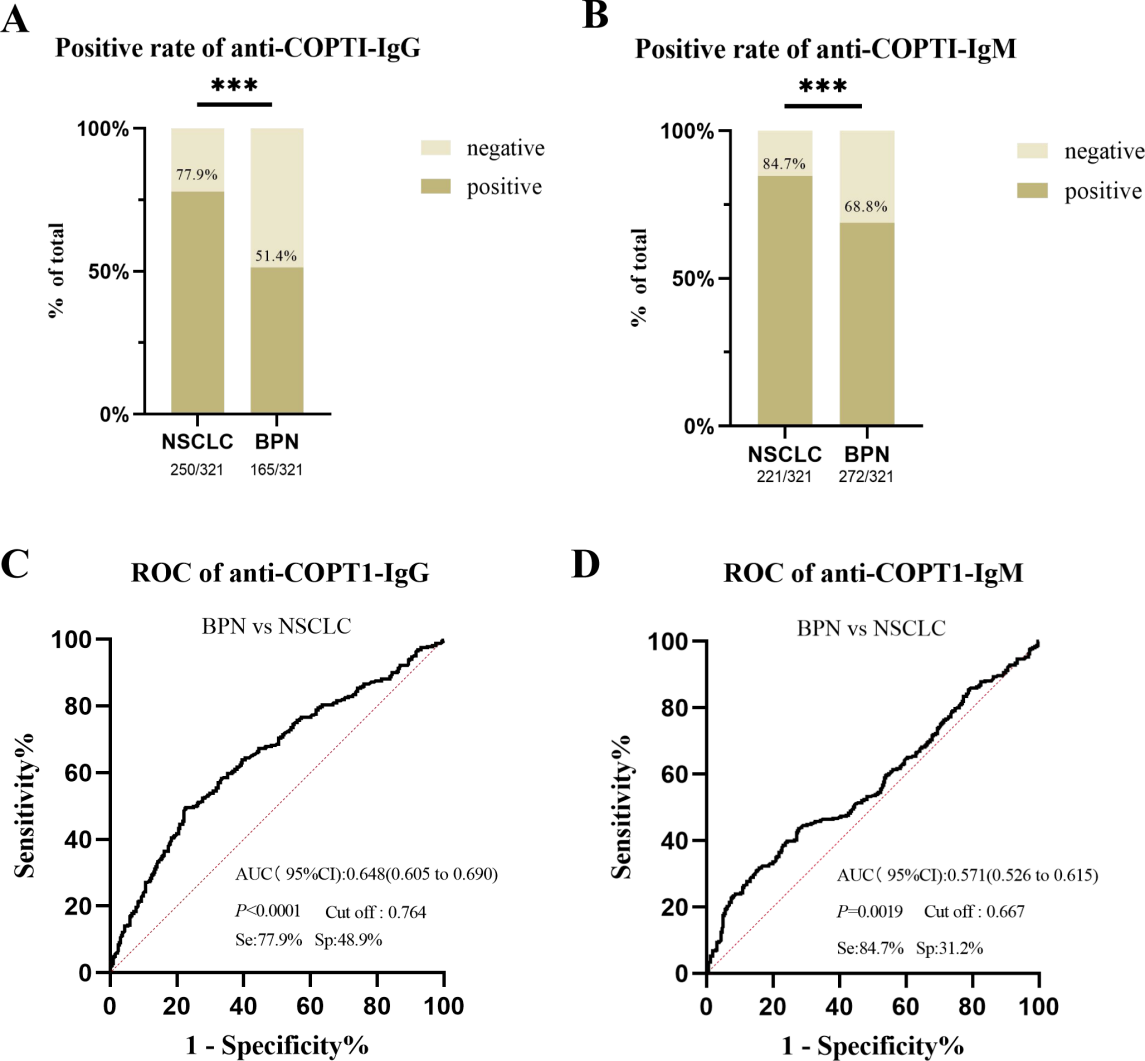
Anti-COPT1 autoantibodies can effectively distinguish NSCLC from BPN. **(A, B)** The positive rates of anti-COPT1-IgG and anti-COPT1-IgM in NSCLC and BPN, respectively. **(C, D)** ROC curve analysis of anti-COPT1 autoantibodies to distinguish NSCLC from BPN. Se, Sensibility; Sp, Specificity. ***P<0.001.

The positive rates of anti-COPT1 autoantibodies in 321 NSCLC and 321 BPN were compared. The positive rates of anti-COPT1-IgG and anti-COPT1-IgM autoantibodies were higher in NSCLC than in BPN (**Figures 8A, B**) (*P*<0.05). The AUC values of anti-COPT1-IgG and anti-COPT1-IgM were 0.648 (95% CI: 0.605-0.690, sensitivity=77.9%, specificity=48.9%) and 0.571 (95% CI: 0.526-0.615, sensitivity=84.7%, specificity=31.2%), respectively (**Figures 8C, D**). The results indicated that anti-COPT1-IgG and anti-COPT1-IgM autoantibodies could distinguish NSCLC from BPN.

### Anti-COPT1 autoantibodies can distinguish NSCLC clinical subgroups from BPN

**Figure 9:**
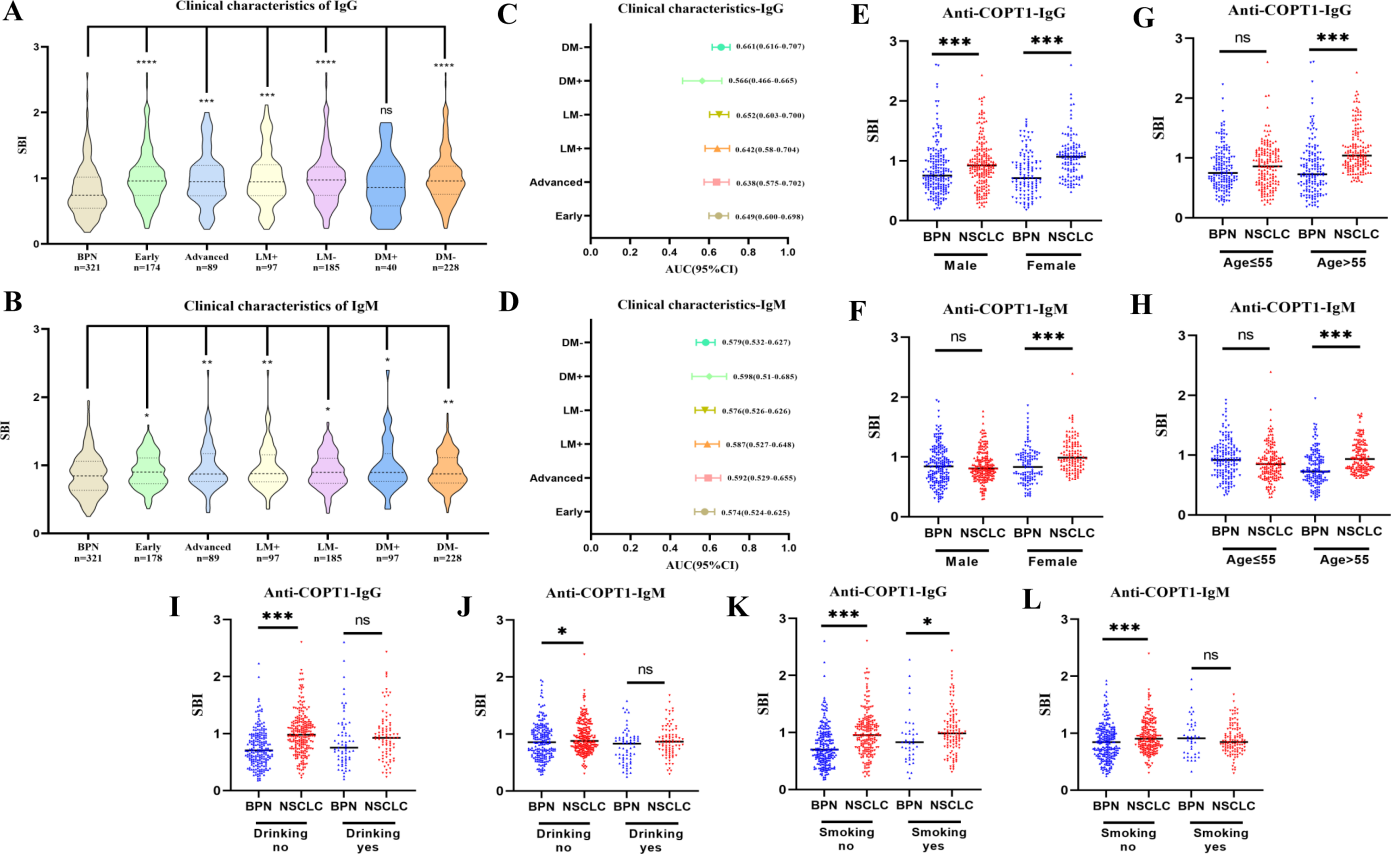
Expression of anti-COPT1 in different clinical subgroups and BPN. **(A, B)** Analysis of the expression of anti-COPT1 autoantibodies in different clinical subgroups and BPN, respectively. **(C, D)** AUC value and 95% CI of anti-COPT1 autoantibodies to distinguish NSCLC with different clinical characteristics from BPN. **(E–L)** The expression of anti-COPT1-IgG and anti-COPT1-IgM autoantibodies stratified by age, gender, smoking and drinking status. Early, Patients with early NSCLC (clinical stage I&II); Advanced, Patients with advanced NSCLC (clinical stage III&IV); LM+, Lymph node metastasis positive; LM-, Lymph node metastasis negative; DM+, Distant metastasis positive; DM-, Distant metastasis negative; Smoking yes/no, Individuals with or without smoking history; Drinking yes/no, Individuals with or without drinking history. *P<0.05, **P<0.01, ***P<0.001.

The expression of anti-COPT1-IgG autoantibodies in different clinical subgroups except for the NSCLC subgroup with distant metastasis, was higher than that in BPN (**Figure 9A**). The expression level of anti-COPT1-IgM autoantibody in NSCLC patients with a history of drinking and smoking was not statistically different from that in BPN (*P*>0.05) (**Figure 9B**). The AUC of the different subgroups was obtained (**Figures 9C, D**). The AUC value of anti-COPT1-IgG in NSCLC with different clinical features ranged from 0.566 to 0.661 (**Figure 9C**), while the AUC value of anti-COPT1-IgM in NSCLC with different clinical features ranged from 0.524 to 0.598 (**Figure 9D**). Anti-COPT1-IgG and anti-COPT1-IgM autoantibodies discriminated early NSCLC from BPN, with AUC values of 0.649 and 0.574, respectively (**Figures 9C, D** **).**

Pairwise comparisons of the subgroups stratified by age, gender, smoking status and drinking status were performed. In females, anti-COPT1 autoantibody levels were higher in NSCLC than in BPN (**Figures 9E, F**). The AUC values of anti-COPT1-IgG and anti-COPT1-IgM to distinguish NSCLC from BPN in females was 0.720 (95% CI: 0.655-0.784, sensitivity=60.7%, specificity=74.4%) and 0.682 (95% CI: 0.615-0.749, sensitivity=86.9%, specificity=47.1%), respectively (**Supplementary Figures 4A, B**). For individuals aged >55 years, the AUC values of anti-COPT1-IgG and anti-COPT1-IgM were 0.745 (95% CI: 0.690-0.800, sensitivity=94.3%, specificity=50.0%) and 0.713 (95% CI: 0.656-0.770, sensitivity=82.2%, specificity=55.8%), respectively (**Figures 9G, H**, **Supplementary Figures 4C, D**). Stratified by smoking and drinking status, the anti-COPT1-IgG levels were higher in NSCLC without smoking and drinking history, and the AUC values for the subgroups increased (**Figures 9I–L**, **Supplementary Figures 4E–H**). Anti-COPT1-IgG autoantibody had a higher diagnostic value (AUC=0.707, 95% CI: 0.659-0.756, sensitivity=79.7%, specificity=53.6%) for distinguishing NSCLC without drinking history from BPN (**Supplementary Figure 4E**).

### Combination of anti-COPT1-IgG and anti-COPT1-IgM improved the diagnostic value

**Figure 10:**
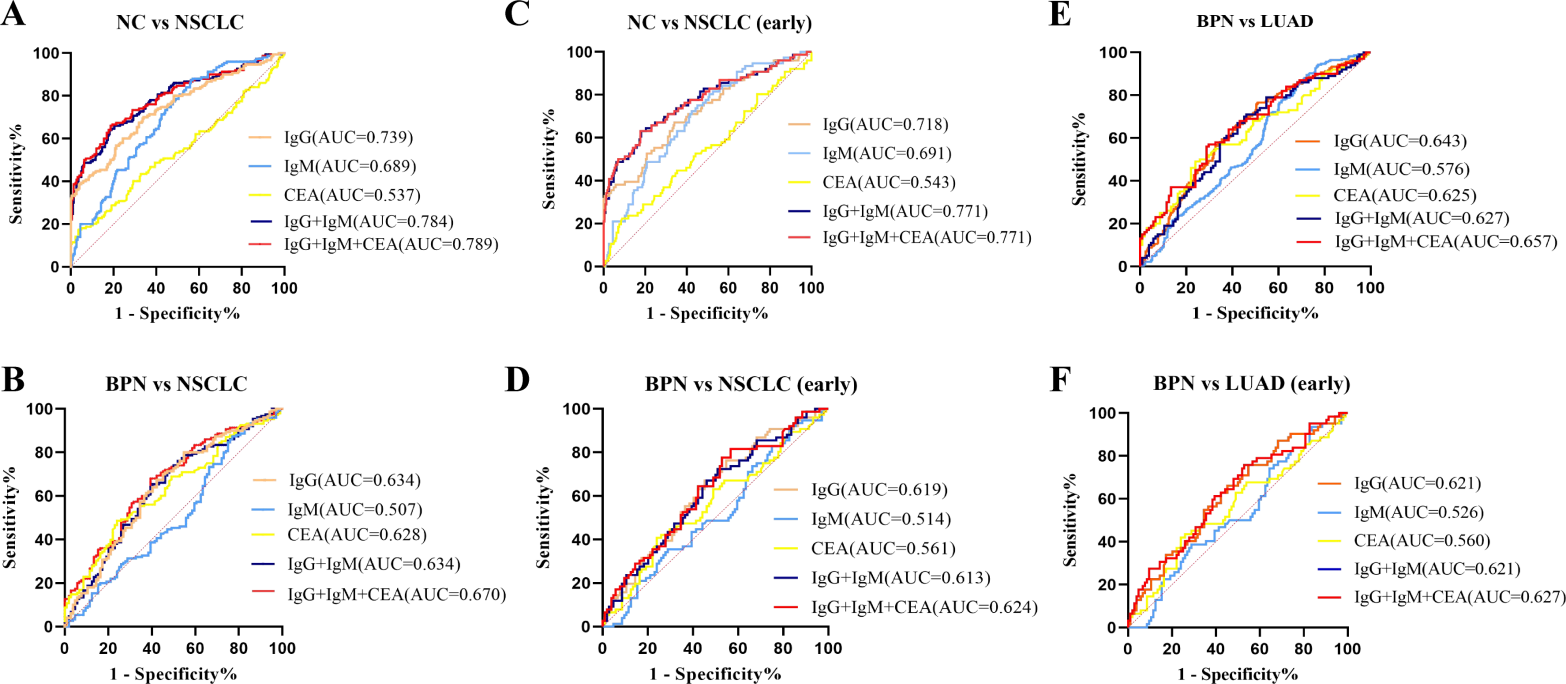
Combined diagnosis of anti-COPT1 autoantibodies and CEA to evaluate the diagnostic value. **(A)** Combined diagnosis of anti-COPT1 autoantibodies and CEA to distinguish NSCLC from NC. **(B)** Combined diagnosis of anti-COPT1 autoantibodies and CEA to distinguish NSCLC from BPN. **(C)** Combined diagnosis of anti-COPT1 autoantibodies and CEA to distinguish early NSCLC from NC. **(D)** Combined diagnosis of anti-COPT1 autoantibodies and CEA to distinguish early NSCLC from BPN. **(E)** Combined diagnosis of anti-COPT1 autoantibodies and CEA to distinguish LUAD from BPN. **(F)** Combined diagnosis of anti-COPT1 autoantibodies and CEA to distinguish early LUAD from BPN. IgG, Anti-COPT1-IgG autoantibody; IgM, Anti-COPT1-IgM autoantibody.

In the verification group, samples with expression information for the traditional tumor marker CEA were selected for multiplex biomarker assays. The combined diagnostic value of anti-COPT1-IgG, anti-COPT1-IgM and CEA in 226 NC, 150 NSCLC and 105 BPN were analyzed to examined the additional information on NSCLC diagnosis. The results indicated that the combined diagnosis of anti-COPT1-IgG and anti-COPT1-IgM autoantibodies can improve the diagnostic value (AUC=0.784, 95% CI: 0.736-0.833) for distinguishing NSCLC from NC (**Figure 10A**). The accuracy of biomarker testing was increased by the combinational analysis (**Table 3**). However, combined diagnosis with CEA did not significantly improve the diagnostic value (**Figure 10A**). The combination of anti-COPT1-IgG, anti-COPT1-IgM, and CEA enhanced the efficacy of NSCLC diagnosis from BPN, and the AUC value increased up to 0.670 (95% CI: 0.603-0.737) (**Figure 10B**, **Table 4**). Moreover, the combination models improved the diagnostic value for distinguishing early NSCLC from NC and BPN, with the AUC values increasing up to 0.771 (95%CI: 0.703-0.883, sensitivity=63.2%, specificity=82.3%) and 0.624 (95% CI: 0.541-0.706, sensitivity=81.6%, specificity=43.3%), respectively (**Figures 10C, D**, **Table 4**). Finally, we performed the combined analysis to distinguish LUAD from BPN (**Figures 10E, F**). The results showed that this combination can improve the diagnostic ability of LUAD (AUC=0.657, 95% CI: 0.548-0.702), especially for early LUAD (AUC=0.627, 95% CI: 0.540-0.715).

**Table 3:** Diagnostic efficacy of anti-COPT1 autoantibodies combined with CEA in NSCLC vs NC.

		AUC	95% CI	Se (%)	Sp (%)	AR (%)
NC vs NSCLC	IgG IgM CEA IgG+IgM IgG+IgM+CEA	0.739 0.689 0.537 0.784 0.789	0.686-0.791 0.636-0.742 0.476-0.599 0.736-0.833 0.740-0.838	70.0 87.3 17.3 64.0 66.0	65.9 43.8 96.5 81.9 81.4	56.7 61.2 64.9 74.7 75.0
NC vs NSCLC (Early)	IgG IgM CEA IgG+IgM IgG+IgM+CEA	0.718 0.691 0.543 0.771 0.771	0.648-0.788 0.625-0.757 0.465-0.622 0.703-0.838 0.703-0.838	67.1 75.0 22.4 63.2 63.2	65.9 54.9 91.2 81.9 82.3	66.2 59.9 70.8 77.1 77.5

Se, Sensitivity; Sp, Specificity; AR, Agreement rate; CI, Confidence interval; IgG, Anti-COPT1-IgG autoantibody; IgM, Anti-COPT1-IgM autoantibody.

**Table 4:** Diagnostic efficacy of anti-COPT1 autoantibodies combined with CEA in NSCLC vs BPN.

		AUC	95%CI	Sp (%)	Sp (%)	AR (%)
BPN vs NSCLC	IgG IgM CEA IgG+IgM IgG+IgM+CEA	0.634 0.507 0.628 0.634 0.670	0.564-0.705 0.433-0.581 0.559-0.697 0.564-0.705 0.603-0.737	80.0 87.3 48.6 80.0 52.7	45.2 23.1 76.0 45.2 75.5	47.2 61.0 58.7 65.7 61.8
BPN vs NSCLC (Early)	IgG IgM CEA IgG+IgM IgG+IgM+CEA	0.619 0.514 0.561 0.613 0.624	0.537-0.701 0.429-0.599 0.475-0.647 0.531-0.696 0.541-0.706	67.1 92.1 48.6 72.4 81.6	54.8 16.3 76.0 49.0 43.3	59.4 48.3 60.5 58.9 59.4

Se, Sensitivity; Sp, Specificity; AR, agreement rate; CI, Confidence interval; IgG, Anti-COPT1-IgG autoantibody; IgM, Anti-COPT1-IgM autoantibody.

## Discussion

In this study, we identified anti-COPT1 autoantibodies as novel tumor-associated autoantibody biomarkers for NSCLC detection. Our study showed that the expression levels of anti-COPT1-IgG and anti-COPT1-IgM in NSCLC were significantly higher than those in BPN and NC. Anti-COPT1-IgG and anti-COPT1-IgM autoantibodies can effectively distinguish NSCLC from NC with AUC values of 0.733 and 0.679 respectively, and distinguish NSCLC from BPN with AUC value of 0.648 and 0.571 respectively. The combined diagnosis of anti-COPT1-IgG and anti-COPT1-IgM improved the diagnostic value for distinguishing NSCLC from NC, and the AUC values increased to 0.784. When distinguishing NSCLC from BPN, the combined diagnosis of anti-COPT1-IgG, anti-COPT1-IgM and CEA enhanced the accuracy of NSCLC detection with AUC of 0.670.

Over the past decade, tissue and blood biomarkers have been identified for NSCLC detection and treatment decisions (24, 25). Plasma-based assays offer numerous advantages over tissue-based assays owing due to their non-invasive nature, rapidity, and ease of repeatability over time. There are various tissue and blood-based assays for biomarker detection, each with its own strengths and limitations (4). Various TAAs have been identified in almost all types of cancers. Many antigens found in blood have been evaluated as potential biomarkers of lung cancer. The most studied biomarkers include CYFRA21-1, CEA, neuron Specific Enolase (NSE), and squamous cell carcinoma antigen (SCC-Ag). CEA is a cell surface glycoprotein produced during fetal development that plays a crucial role in cell adhesion. Elevated CEA levels have been linked to various cancers, such as lung, colorectal, and thyroid cancers (26). Increased serum CEA levels are frequently observed in NSCLC patients, particularly those with adenocarcinoma (27, 28). However, the diagnostic value of CEA alone for lung cancer is limited due to its low sensitivity and specificity (29, 30). Tumor-associated autoantibodies are antibodies produced by the immune response against various TAAs, such as overexpressed antigens, mutated proteins, or post-translationally modified proteins (31). Some autoantibodies have high potential as biomarkers due to their sensitivity and specificity (32).

As commonly understood, copper is a crucial cofactor for all organisms, including the human body (16). When copper concentrations exceed the normal threshold, it becomes toxic and causes cell death, known as cuproptosis (33). Abnormal regulation of some cuproptosis-related genes has been shown to play an important role in the occurrence and progression of cancers (34–36). Zhang et al. (37), comprehensively examined the carcinogenicity of COPT1 in various cancer types, and demonstrated that COPT1 may be an effective biomarker for cancer prognosis. COPT1 expression correlated with the expression of PD-L1 and the infiltration of immune cell infiltration, indicating its potential significance in tumor treatment (38, 39). In addition, some studies found that COPT1 is a potential copper death-related gene in breast cancer, which is significantly upregulated, and has great potential for predicting the prognosis, diagnosis and drug sensitivity of breast cancer (21). Based on TIMER and HPA databases, the expression level of COPT1 was higher in NSCLC and correlated with various infiltrated immune cells (**Supplementary Figure 5**). COPT1 mutations were identified in multiple tumors, including lung cancer with a mutation rate of 1.5% (40). High expression of COPT1 and COPT1 mutations may trigger strong immune responses to COPT1 autoantibodies. In addition, COPT1 expression was positively correlated with infiltrated CD4^+^ T, CD8^+ ^T, and B cells. These results suggest that COPT1 is a TAA and induced the increase of anti-COPT1 autoantibodies in NSCLC patients. However, elevated COPT1 expression were reported in various tumors, indicating that anti-COPT1 autoantibodies may be induced in other tumors. The clinical value of anti-COPT1 autoantibodies for the detection of other cancers and their specificity for NSCLC should be elucidated in future studies.

Autoantibodies play a significant role in the diagnosis and prognosis of lung cancer. Certain autoantibodies exhibit promising diagnostic capabilities, and studies have indicated that serum autoantibodies targeting GAGE7, MAGEA1, CAGE, and p53 can aid in the diagnosis of lung cancer. Immunoglobulin G (IgG) and immunoglobulin M (IgM) autoantibodies are important components produced by humoral immune reactions and secreted into the blood. Many studies have shown that cancer patients can produce humoral immune responses and then produce autoantibodies at an early stage before cancer diagnosis (41). Therefore, IgM and IgG autoantibodies, as the first and second reaction products of humoral immunity, have great potential as early diagnostic indicators of cancer (42). Currently, research on screening autoantibody biomarkers for the diagnosis and treatment of lung cancer primarily forced on IgG autoantibodies (43–45). However, IgM autoantibodies, as the first antibodies produced by immune response, may be more suitable for screening indicators for early cancer diagnosis (46). A study shows that (36), IgM autoantibodies are crucial for the immune surveillance against malignant epithelial cells. In this study, the levels of both anti-COPT1-IgG and anti-COPT1-IgM autoantibodies in NSCLC, BPN and NC were detected by ELISA, and combined with the traditional tumor marker CEA to evaluate the diagnostic value. The expression of anti-COPT1 autoantibodies were higher in NSCLC. Anti-COPT1-IgG and anti-COPT1-IgM autoantibodies were demonstrated to be preferable biomarkers for NSCLC detection. Anti-COPT1-IgG performed a higher power for NSCLC detection than anti-COPT1-IgM. Moreover, the combined detection of anti-COPT1 autoantibodies and CEA in patient plasma improved the accuracy of NSCLC diagnosis.

Our study demonstrated that anti-COPT1 autoantibodies have the potential to enhance the early detection of NSCLC, thereby improving patient outcomes. However, limitations such as the small sample size and reliance on a single sample source need to be addressed. Large-sample, multicenter studies are warranted to further validate these findings. Sensitivity and specificity are basic statistical principles for assessing the performance of diagnostic tests. In the present study, the sensitivity and specificity were not particularly high. Population diversity including age, gender and ethnicity may affect the generalizability of the findings. Thus, it now seems to be premature to define anti-COPT1 autoantibodies combined with CEA as cancer screening or early stage cancer biomarkers for clinical application. Further validation studies involving large-scale, well-defined cohorts with diverse populations and comprehensive clinical data are warranted to confirm the diagnostic potential of anti-COPT1 autoantibodies. Additionally, comprehensive combination analysis integrating anti-COPT1 autoantibodies with other conventional tumor markers (e.g., AFP, CA199, CA125) are recommended to enhance their clinical utility and diagnostic accuracy. Retrospective or prospective studies should conducted to verify its usefulness for cancer diagnosis and prognosis in the future study.

Furthermore, to enhance the translational potential of anti-COPT1 autoantibodies for NSCLC detection, the functional studies should be performed to investigate the mechanism of action of anti-COPT1 autoantibodies in NSCLC progression.

In summary, this is the first study to report anti-COPT1 autoantibodies in NSCLC. Anti-COPT1 autoantibodies were highly expressed in NSCLC and could distinguish NSCLC from NC and BPN. Both IgG and IgM autoantibodies against COPT1 present the diagnostic value for the patients with NSCLC at early stage. The combination of anti-COPT1 autoantibodies and CEA had contributed to the further improvements in the early diagnosis for NSCLC. Our study indicated that anti-COPT1 autoantibodies can be used as potential novel plasma biomarkers for detecting NSCLC.

## Data Availability

The raw data supporting the conclusions of this article will be made available by the authors, without undue reservation.
