# Supplementary material for: Plasma IgG and IgM autoantibodies to COPT1 as potential biomarkers for detection of non-small cell lung cancer
Source: Front Immunol. 2025 Apr 11;16:1455095. doi: 10.3389/fimmu.2025.1455095 (PMC12021867; doi:10.3389/fimmu.2025.1455095)
Supplement: Supplementary file 1 [file DataSheet1.pdf]

Supplementary Figures

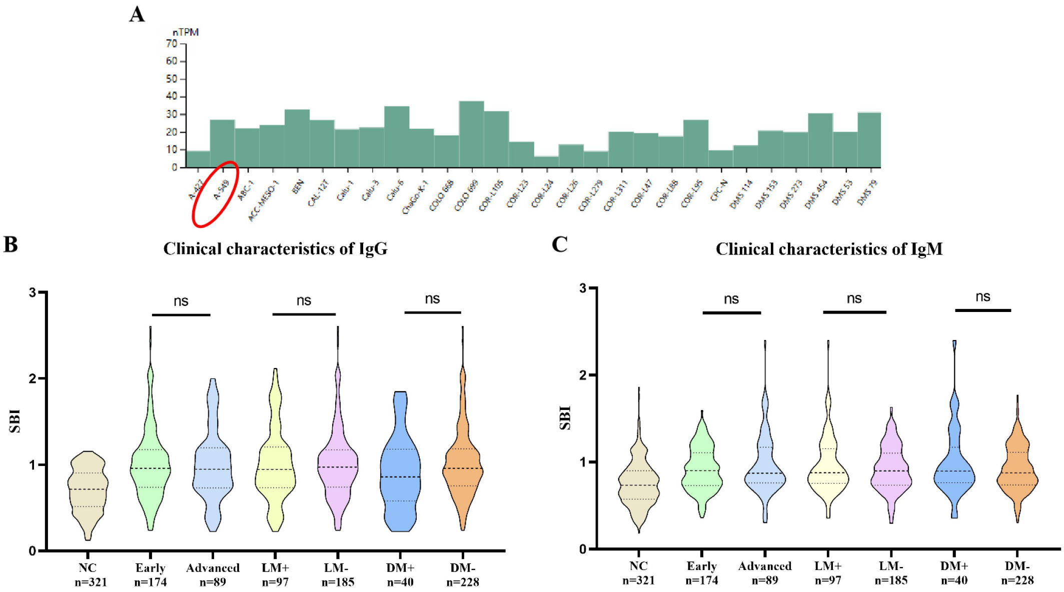

Supplementary Figure 1. The expression of anti-COPT1 in different clinical subgroups and COPT1 in lung cancer cell lines.

A. The expression of COPT1 in different lung cancer cell lines on GEPIA database. B, C. Expression of anti-COPT1-IgG and anti-COPT1-IgM autoantibodies in different clinical subgroups, respectively.

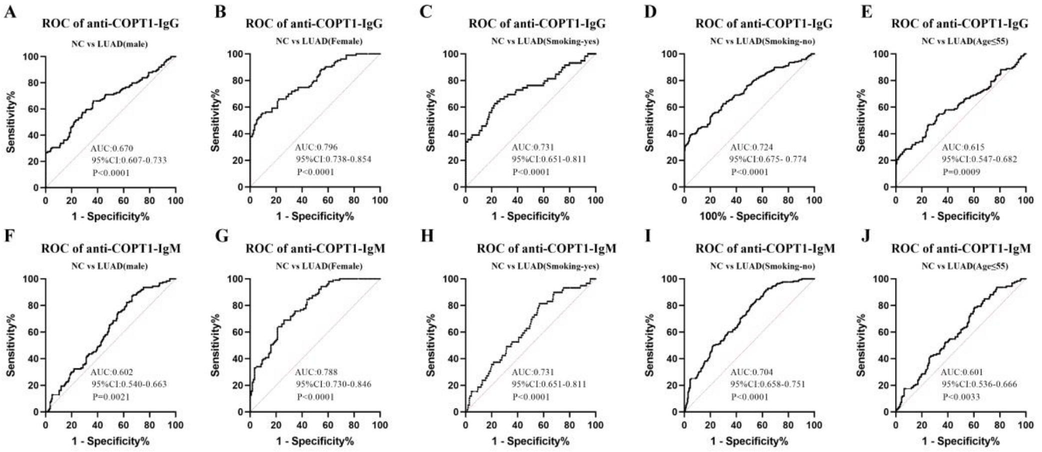

Supplementary Figure 2. Diagnostic efficacy of anti-COPT1 autoantibodies in LUAD with different clinical features. Clinical features including Male (A), (F); Female (B), (G); Smoking (C), (H); Smoking-no (D), (I); Age≤55 (E), (J) .

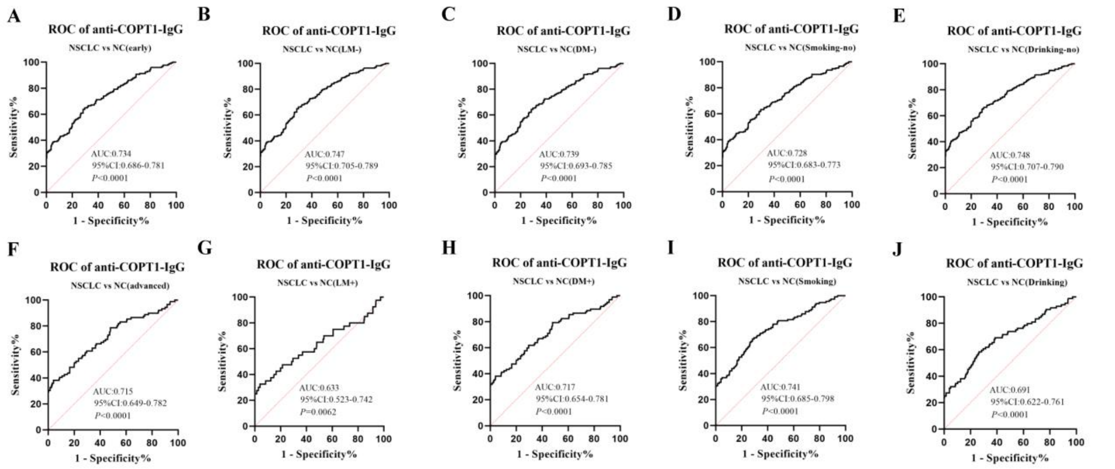

Supplementary Figure 3. Diagnostic efficacy of anti-COPT1-IgG in different clinical subgroups. Clinical subgroups including Early (A), Advanced (B), LM-(C), LM+ (D), DM-(E), DM+ (F), nonsmoking (G), Smoking (H), nondrinking (I), Drinking (J). LM, Lymph node metastasis; DM, Distant metastasis. Early, Patients with early NSCLC (clinical stage I&II); Advanced, Patients with advanced NSCLC (clinical stage III&IV); LM+, Lymph node metastasis positive; LM-, Lymph node metastasis negative; DM+, Distant metastasis positive; DM-, Distant metastasis negative

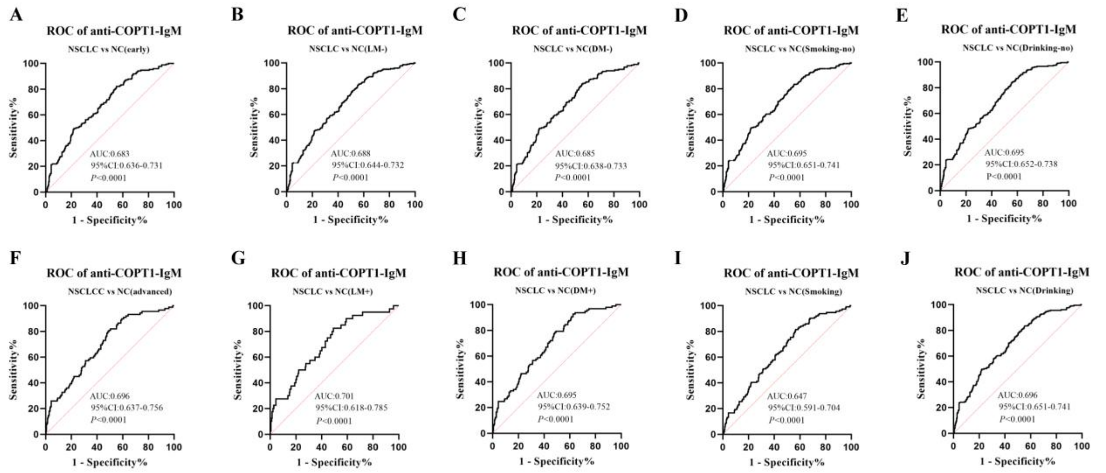

Supplementary Figure 4. Diagnostic efficacy of anti-COPT1-IgM in different clinical subgroups. Clinical subgroups including Early (A), Advanced (B), LM-(C), LM+ (D), DM-(E), DM+ (F), nonsmoking (G), Smoking (H), nondrinking (I), Drinking (J). LM, Lymph node metastasis; DM, Distant metastasis. Early, Patients with early NSCLC (clinical stage I&II); Advanced, Patients with advanced NSCLC (clinical stage III&IV); LM+, Lymph node metastasis positive; LM-, Lymph node metastasis negative; DM+, Distant metastasis positive; DM-, Distant metastasis negative

A

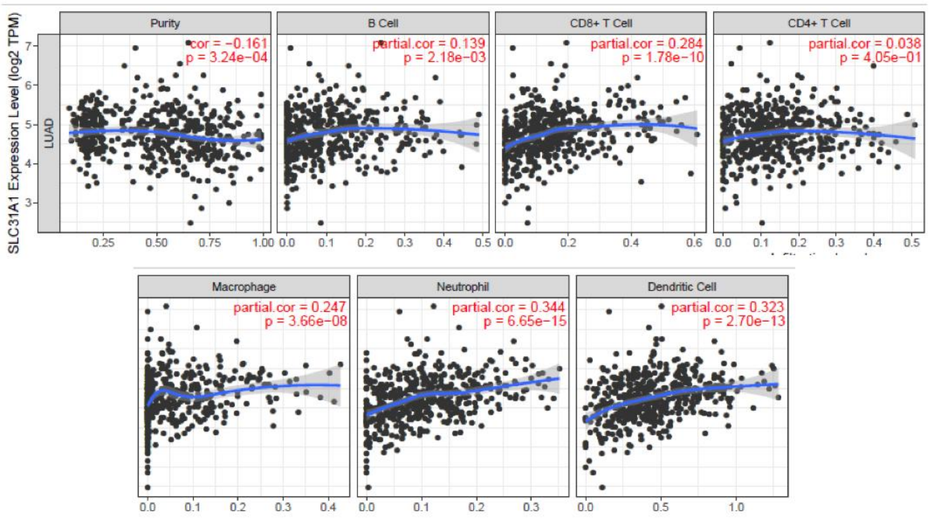

B

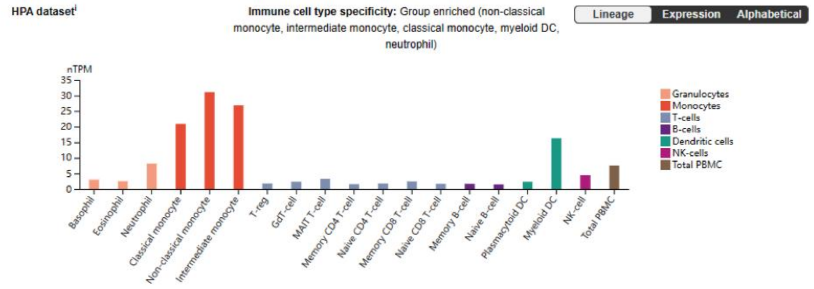

Supplementary Figure 5. Correlation between COPT1 expression and various immune cells. Through TIMER (A) and HPA (B) database, the correlation between the expression level of COPT1 and the infiltration of immune cells was analyzed.
